# Climatic, environmental factors and agricultural practices favoring dynamics of the spread of African cassava mosaic disease in Côte d’Ivoire

**DOI:** 10.1007/s44279-026-00550-2

**Published:** 2026-03-12

**Authors:** Bekanvié S. M. Kouakou, John Steven S. Seka, Justin S. Pita, Aya Ange Naté Yoboué, Israël Tankam Chedjou, Guy Roland Eboulem, Nazaire K. Kouassi, Fidèle Tiendrébéogo, Fatogoma Sorho

**Affiliations:** 1 UFR Biosciences, Université Félix Houphouët-Boigny (UFHB), Abidjan, Côte d’Ivoire; 2 Pôle Scientifique et d’Innovation, The Regional Center of Excellence, Central and West African Virus Epidemiology (WAVE) for Food Security, Université Félix Houphouët-Boigny (UFHB), Abidjan, Côte d’Ivoire

**Keywords:** Cassava mosaic disease, Climatic factors, Altitude, Cropping system, Field density

## Abstract

Cassava mosaic disease (CMD), caused by begomoviruses, poses a major threat to cassava leading to huge yield losses. We analyzed climatic (temperature, humidity, rainfall), non-climatic (altitude, field density, cropping system) variables and also the susceptibility of cassava varieties grown in each agroecological zone, to understand their joint influence on CMD’s spatiotemporal spread in Côte d’Ivoire. Results indicated that all factors interacted to shape CMD epidemiology, but altitude, field density and cropping system showed the strongest effects (*P* < 0.05). Whitefly [Bemisia tabaci] abundance declined with elevation. CMD incidence and symptom severity increased significantly with field density and were higher in intercropping systems, whereas whiteflies thrived in cassava monocultures. Of the climatic parameters analyzed, the most significant correlation was found between temperature and CMD symptom severity which were negatively linked. Humidity and rainfall exerted moderate positive effects on disease levels. The southern areas, with relatively high relative humidity and generally abundant rainfall were found to be most affected by CMD with severe symptoms. The study also showed that local susceptible cassava varieties were more frequently cultivated in the different agroecological zones studied compare to improved cassava varieties that are known more tolerant or resistant to the disease. This may explain why CMD incidence was relatively high in almost all agroecological zones. These results highlight the importance for breeding programs to integrate climatic conditions and cultural practices into targeted CMD management strategies. We encourage the implications of all stakeholders in the agriculture sector to increase their campaign disease surveillance strategies.

## Introduction

Agricultural productivity is often considered as a key factor determining food security in Africa and elsewhere in the tropics. Cassava (*Manihot esculenta* Crantz) is one of the most important staple food for over 500 million people in sub-Saharan Africa [1]. Globally, its production reached 334 million tonnes in 2023, with Africa accounting for 213 million. It is a perennial woody shrub with an edible root, which grows in tropical and subtropical areas of the world [2]. Cassava has the ability to grow in a wider range of climatic conditions and soil types than other tropical staple crops [3]. It can be processed into several end products both by food industries (e.g. pellets, chips, flour, sweetener etc.) and non-food industries (e.g. bio-ethanol, glue) [4]. It is very rich in carbohydrates and can be produced all year round [5]. In Côte d’Ivoire, cassava is produced on three-quarters of the country’s landmass and is the second most important root and tuber crop after yam [6]. All these things demonstrate the crucial role that cassava plays in the socio-economic, industrial and nutritional well-being of the local population. Despite its great importance, cassava production is threatened by several diseases, of which African cassava mosaic disease (CMD) remains the most important for the crop in West Africa. CMD can greatly reduce cassava roots yields by 25–95% [7]. It is a viral disease caused by begomoviruses (Family, *Geminiviridae*: Genus, *Begomovirus*). In Côte d’Ivoire, [8] found ACMV and EACMCMV infecting cassava field with a gradual emergence of EACMCMV in new production areas. Several studies have been conducted to understand CMD epidemiology [8–15] in the country. However, only a few of these studies have demonstrated the factors involved in the spread of the disease. Some studies showed that the increase in disease incidence is directly related to the number of adult whiteflies present in a field and that the highest incidence of African cassava mosaic virus (ACMV) occurred in fields with the lowest plant density. [9, 16] highlighted that CMD incidence and symptom severity varied with cassava cultivars and increased with plant age and climatic conditions. On the other hand, several environmental factors could contribute to the emergence and the development of cassava mosaic disease. These include agronomic practices, cropping system (intercropping or monoculture) [17], field altitude [18]. Moreover, quality of planting material must be considered in setting up a cassava field. Indeed, farmers generally use planting materials from their own fields or from their neighbors, regardless of the phytosanitary status of these planting materials, thus spreading the disease [19]. Furthermore, [20] showed that CMD incidence and symptoms severity vary from one variety to another with plant age. Another important factor that could contribute to disease development is climate conditions [21]. Changes in temperature and rainfall regimes due to climate variation may alter the growth stage, developmental rate, pathogenicity of infectious agents, the physiology and resistance of the host plant [22]. In light of the above, the following research questions were identified:

- What are the main factors influencing the spread of CMD in Côte d’Ivoire?
- How they interact to influence the spread of this disease in the country?

Considering the lack of recent information on the factors driving the spread of CMD in Côte d’Ivoire, we undertook this study to unveil the key factors influencing the epidemiology of this disease in Côte d’Ivoire in order to guide the development of the appropriate measures to control its spread in the country and ensure sustainable productivity of cassava and food security for the Ivorian people.

## Materials and methods

### Field surveys and study site

Field surveys were conducted in Côte d’Ivoire during three years in 2016, 2017 and 2020 [8]. The country is located between 4° and 10° North latitude and between 8° and 9° West longitude. The climate is a transition between equatorial and tropical, generally hot and humid. The country generally experiences significant temperature variations between the North and the South, throughout the year. Temperature average fluctuates between 22 °C and 33 °C. Precipitation is more abundant on the coastal regions, where it ranges from 1,500 to 2,500 mm per year, while inland, it is generally less intense, ranging from 1,200 to 1,500 mm, although it reaches 2,000 mm in the small western mountainous area. Although surveys were conducted across all seven of the country’s agroecological zones, only six were retained for analysis. The seventh zone was excluded due to an insufficient number of cassava fields, making statistical comparisons with the other zones unviable. These zones were identified and defined by [23] according to the pedoclimatic conditions presented in Table 1.

**Table 1:** Characteristics of each agroecological zone (AEZ) of Côte d’Ivoire surveyed

AEZ	Characteristics	Altitude (m)	Rainfall (mm)	Temperatures (°C)
1	Southern humid dense forest zone	0-200	100–2500	29
2	Dense western forest zone	~ 1000	1300–1750	23.5
3	Western semi-mountainous forest zone	> 1000	1300–2300	24.5
4	Dense semi-wet and deciduous forest zone	50–300	1300–1750	23.5
5	Transition forest zone	300–600	1300–1750	23.5
6	Transitional wet savanna zone	300–500	1150–1750	26.7

### CMD associated epidemiological data collection

Young cassava fields (3 to 6 months old) selected randomly along motorable roads at approximately 10 km intervals were surveyed. In each field, 30 cassava plants were selected along the two diagonals for CMD incidence, severity, and whitefly population (Bemisia tabaci adults) assessment as described by [24]. Disease incidence was estimated through the percentage of symptomatic plants over total plants observed. For CMD Severity, a scale of 1 to 5 as described by [25] was used where 1 corresponds to an apparently healthy plant and 5 corresponds to very severe mosaic symptoms including leaf deformation. CMD severity mean per field was then calculated by sum of the scores for diseased plants divided by the number of diseased plants. The number of adult whiteflies per plant was also counted on the top 5 younger leaves [26].

### Environmental data collection

Some environmental parameters were considered for each field surveyed. These parameters include the density of cassava fields defined as the number of cassava fields cultivated in a specific area, the age of the fields and the cropping system (monocultures or intercrops). The crops associated with cassava were also recorded. The altitude of each visited locality was recorded and averages were then calculated for the agroecological zones. Moreover, cassava varieties cultivated in each zone were recorded.

### Climatic data collection

Monthly meteorological data especially rainfall, temperature and relative humidity from 2016 to 2020 were used for this study. The data were obtained from weather archives site for the whole world https://www.historique-meteo.net/afrique/cote-d-ivoire/. The averages of the data collected for each climatic parameter were calculated for each year of survey and per agroecological zone.

### Data analysis

All the data generated were analyzed using R software (version 4.1.1, R Development Core Team, 2010). For the analysis of CMD-associated epidemiological parameters, a generalized linear model (GLM) with a Poisson distribution was employed, assuming that the data followed an exponential family distribution. When significant differences were observed between means, the *emmeans* library was used to estimate adjusted marginal means for each factor level, followed by pairwise comparisons with Tukey’s p-value adjustment (α = 0.05). For climatic and environmental factors, variable normality was assessed using the Shapiro-Wilk test. Differences in climatic parameters among agroecological zones for each year were determined using a one-way analysis of variance (ANOVA) with a post-hoc Tukey’s LSD test. A two-way ANOVA with a post-hoc Tukey’s LSD test was used to determine differences in field density across agroecological zones over the years. Spearman’s correlation coefficient was calculated to test the significance of correlations between CMD epidemiological parameters and the analyzed climatic and environmental factors. Correlations were considered significant at a significance level of *p* < 0.05. A factor analysis of mixed data (FAMD) was also performed to examine the correlations between the three CMD epidemiological parameters and climatic factors, and between the three epidemiological parameters and environmental factors. Prior to FAMD, temperature and rainfall data were normalized using “Scale ()” function from the “base” package of R to prevent scaling bias.

## Results

### Spatiotemporal changes in CMD associated epidemiological parameters

**Fig. 1:**
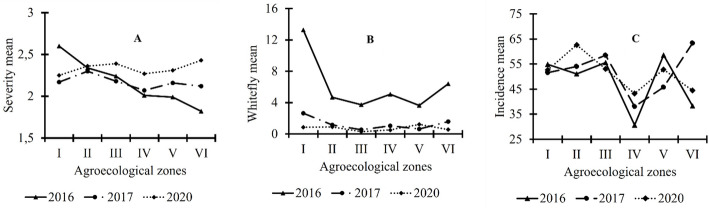
CMD severity mean **A** whitefly mean **B** and Incidence mean **C** showing variations between agroecological zones over the surveyed years

The curve illustrating the evolution of the three CMD associated epidemiological parameters is shown in Fig. 1. The severity of CMD symptoms exhibited variations between the different agroecological zones over the three-year period (Fig. 1A); However, these variations were not statistically significant. As for the whitefly abundance in the study area, the averages were significantly higher in 2016 than in the last two years (Fig. 1B). Regarding CMD incidence between agroecological zones, it is shown that AEZ IV was the least affected zone by CMD over the three-year period (Fig. 1C).

**Fig. 2:**
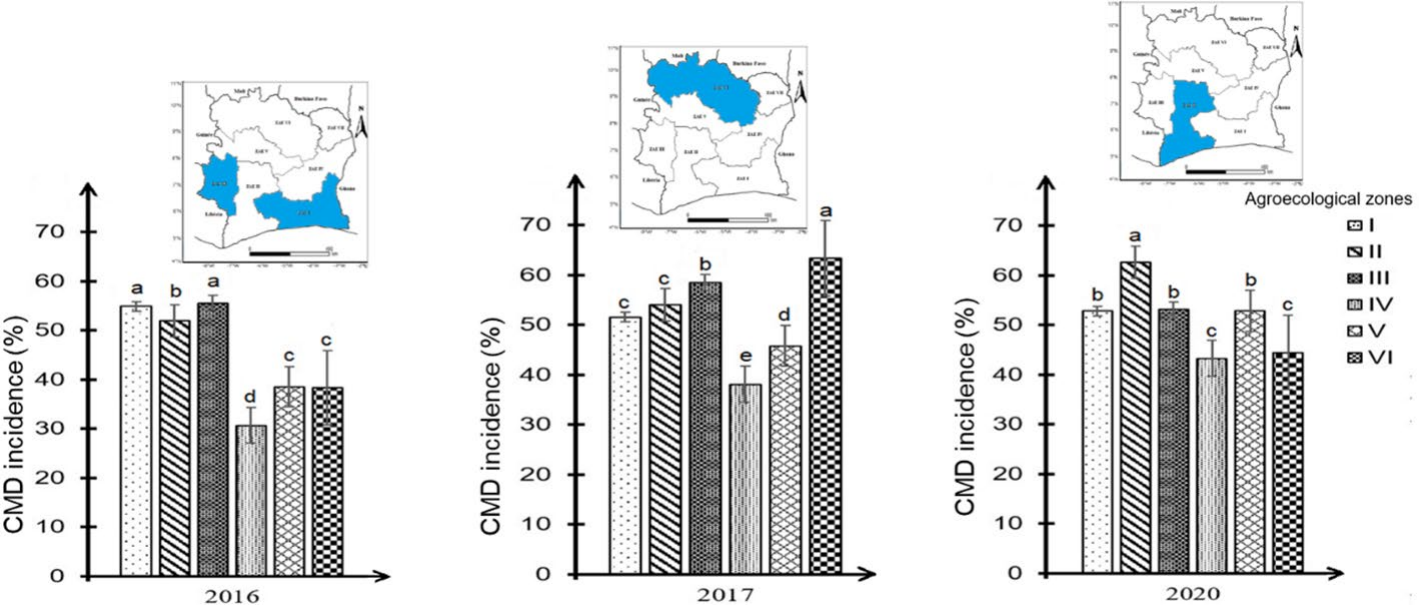
Variation of CMD Incidence between agroecological zones according to the surveyed years in Côte d’Ivoire. Data are means ± SE. The bars represent the standard error. Bars sharing the same letters are not significantly different between years and between agroecological zones (generalized linear model (glm) at 0.05)

In contrast, the AEZs with highest CMD incidence varied significantly over the years. In 2016, the highest incidences were recorded in AEZ I and III. In 2017, it was AEZ VI which recorded the highest incidence mean and during the last year, AEZ II was found to be the most affected by the disease (Fig. 2).

### Spatiotemporal changes in main climatic parameters

**Fig. 3:**
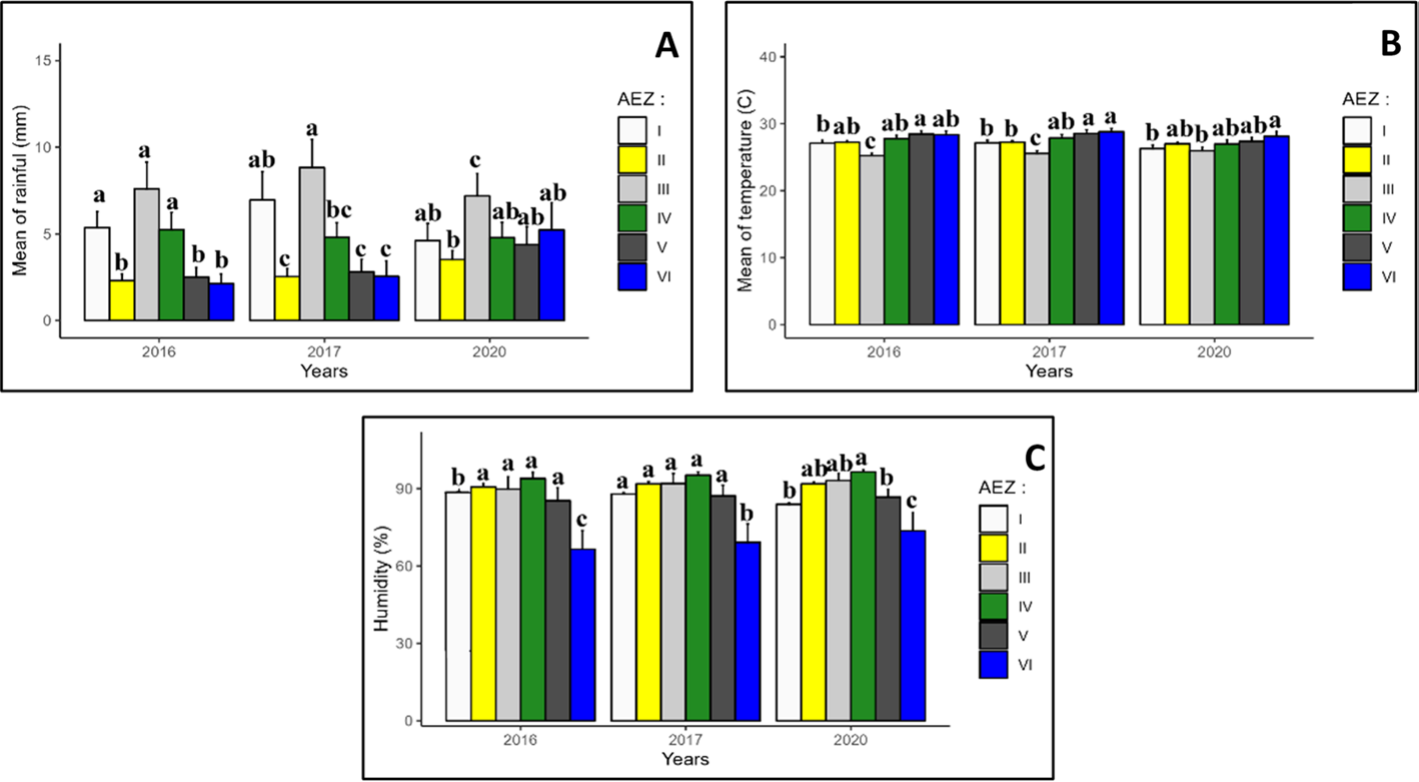
Variations in rainfall **A** temperature **B** and relative humidity **C** between agroecological zones over the surveyed years. Data are means ± SE. The bars represent the standard error. Statistical differences were determined with a one-way ANOVA and Tukey’s LSD test. Bars assigned different letters are significantly different at 0.05 between agroecological zones

Changes in monthly rainfall, temperature and relative humidity between agroecological zones from 2016 to 2020 are shown in Fig. 3. A thorough statistical analysis of the climatic data revealed significant variations in rainfall, temperature and relative humidity between the AEZs. AEZ III experienced the greatest rainfall mean during the three-year period in 2016, 2017 and 2020. However, the lowest average rainfall was recorded by AEZ II, AEZ V and AEZ VI (Fig. 3A). It is notable that AEZ III was characterized by the lowest recorded temperatures (Fig. 3B). With regard to relative humidity, the lowest mean values were recorded in AEZ VI over the three-year period (Fig. 3C).

### Spatiotemporal changes in environmental parameters

**Fig. 4:**
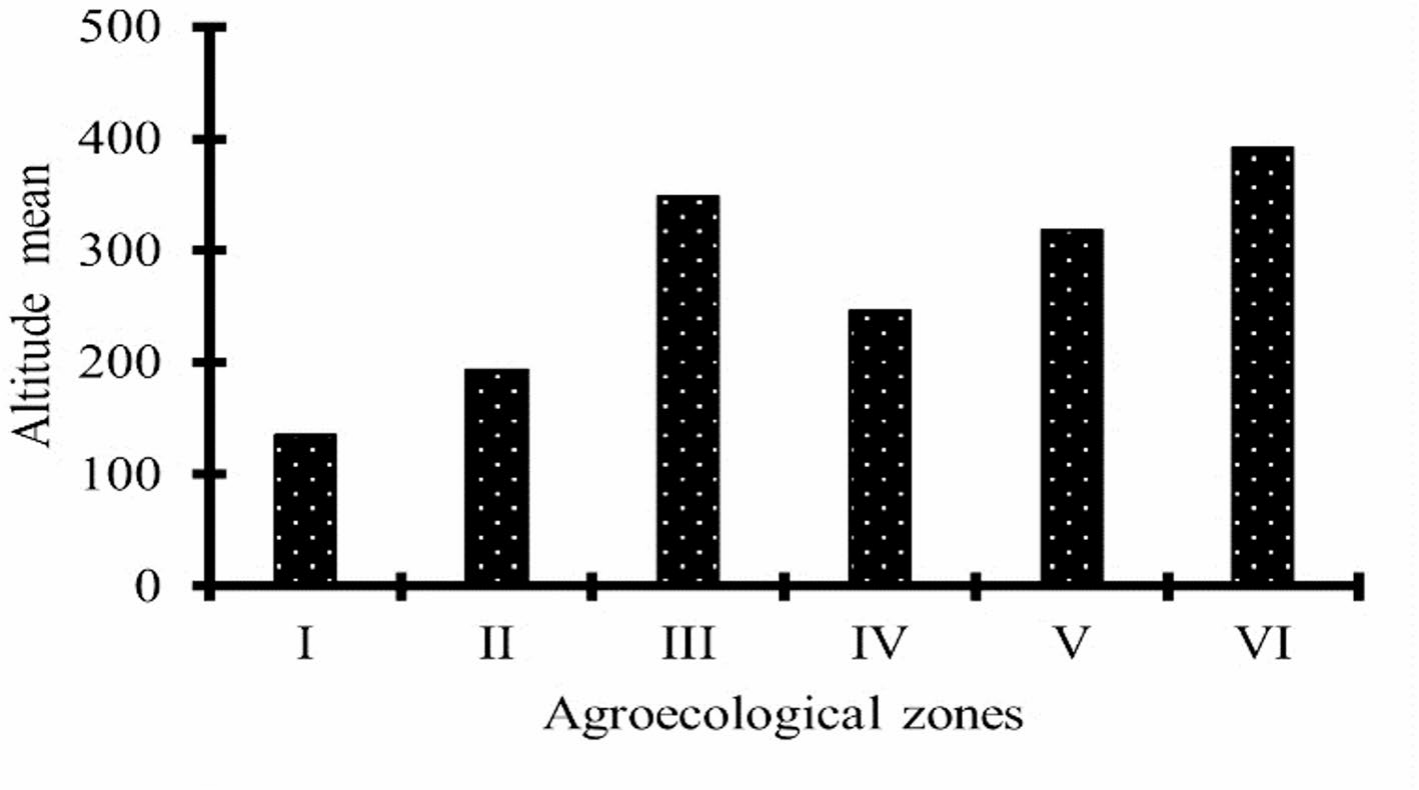
Altitude means according to agroecological zones

The data collected on average altitude and shown in Fig. 4 revealed differences between the agroecological zones. AEZs I and II, located in the southern and center-western Côte d’Ivoire, and AEZ IV in the eastern Côte d’Ivoire, were found to be the low-altitude zones (˂ 300 m). In contrast, AEZs III, V and VI in the west and north part of the country, respectively, recorded the highest altitudes (˃300 m).

**Fig. 5:**
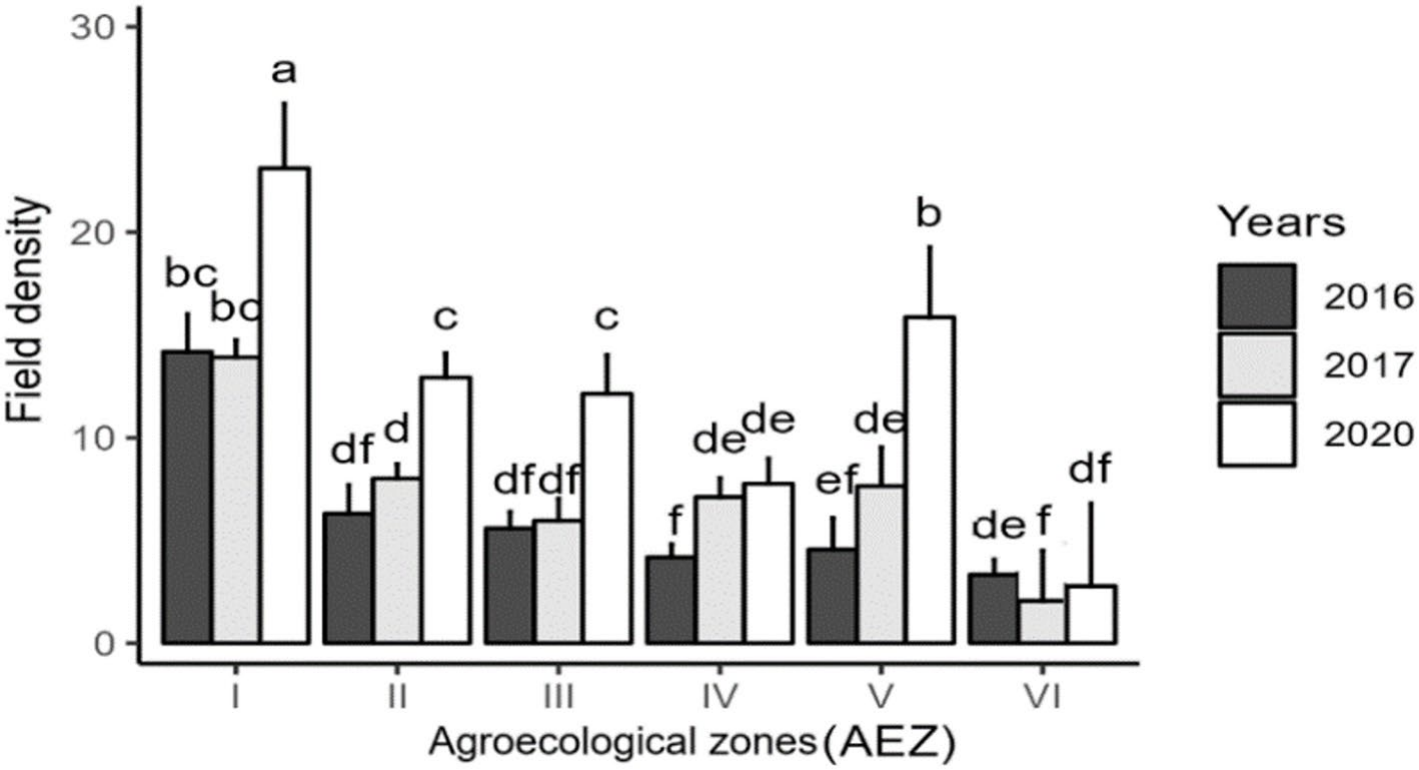
Field density according to agroecological zone over the surveyed years. Data are means ± SE. The bars represent the standard error. Statistical differences were determined with a two-way ANOVA and Tukey’s LSD test. Bars assigned different letters are significantly different at 0.05 between agroecological zones and years

Statistical analysis of cassava field density per zone, shown in Fig. 5 exhibited a highly significant difference (P˂0.001) between AEZs. Cassava fields are unevenly distributed throughout the country. AEZ I is the zone where cassava is most cultivated. In contrast, the other zones exhibited relatively fewer cassava fields compared to AEZ I, with AEZ VI registering the lowest number of fields over the three-year survey period.

**Fig. 6:**
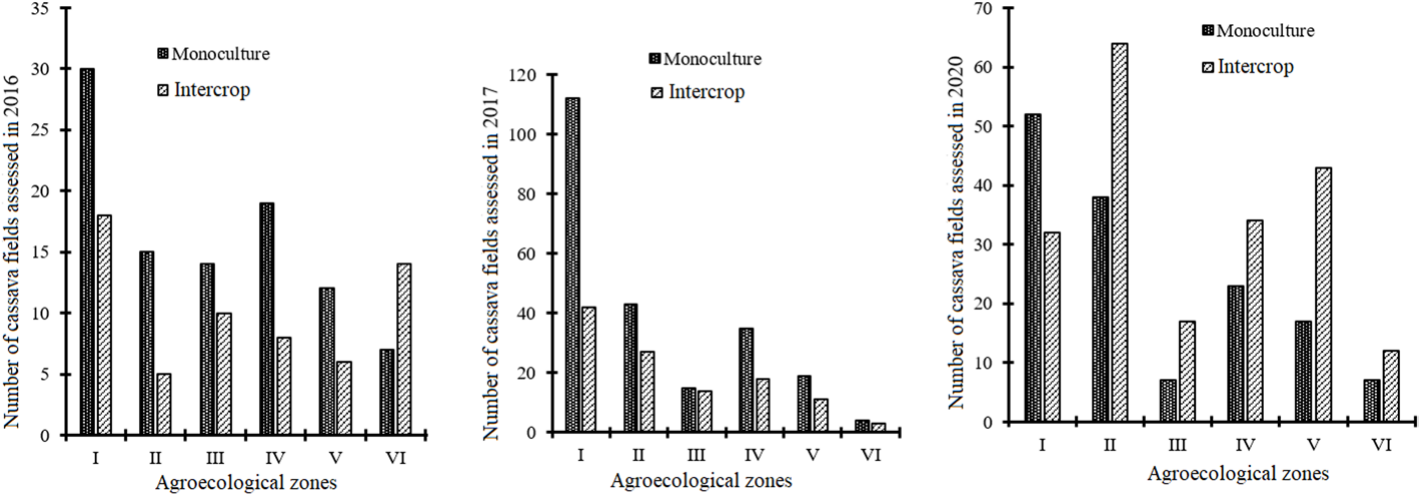
Cropping systems according to agroecological zones over the surveyed years

The results presented in Fig. 6 concerning cropping systems demonstrated that the majority of the assessed fields were monocultures (cassava only, 55.62%) with intercropped cassava fields representing 44.38% of the plots surveyed over the three years. This was not statistically significant. However, highly significant differences were noticed between the AEZs. In 2016, with the exception of AEZ VI, other zones registered more fields being monoculture of cassava compared to those of cassava associated with other crops. A similar scenario was observed in 2017, with the number of fields in both monoculture and intercropping in AEZ VI remaining constant. A marked shift in trend was observed in 2020, wherein all the zones under assessment exhibited a greater prevalence of intercropping relative to cassava monoculture fields, with the exception of Zone I, which exhibited a consistent trend across all three years.

Several crops were found in association with cassava and are presented in Table 2. These include *Musa paradisiaca* (Plantain), *Zea mays* (Maize), *Elaeis guineensis* (Palm tree), *Hevea brasiliensis* (Rubber tree), *Theobroma cacao* (Cocoa), *Colocasia esculenta* (Taro), *Dioscorea spp.* (Yam), *Ipomoea batata* (Sweet potato), *Sorghum bicolor* (Sorghum), *Arachis hypogaea* (Peanut), *Anacardium occidentale* (cashew nuts), *Ananas comosus* (Pineapple), *Capsicum annuum* (Pepper), *Abelmoschus esculentus* (Okra), *Solanum melongena* (Eggplant) and *Solanum lycopersicum* (Tomato). However, *Manihot esculenta* (cassava)/*Musa paradisiaca* (Plantain) and *Manihot esculenta* (cassava)/*Zea mays* (Maize) were the two crop associations most frequently found during the surveys with the rate of 32.7% and 38% respectively. The association *Manihot esculenta* (cassava)/*Musa paradisiaca* (Plantain) was more found in AEZ I and AEZ II while *Manihot esculenta* (cassava)/*Zea mays* (Maize) were more encountered in AEZ V and AEZ VI.

**Fig. 7:**
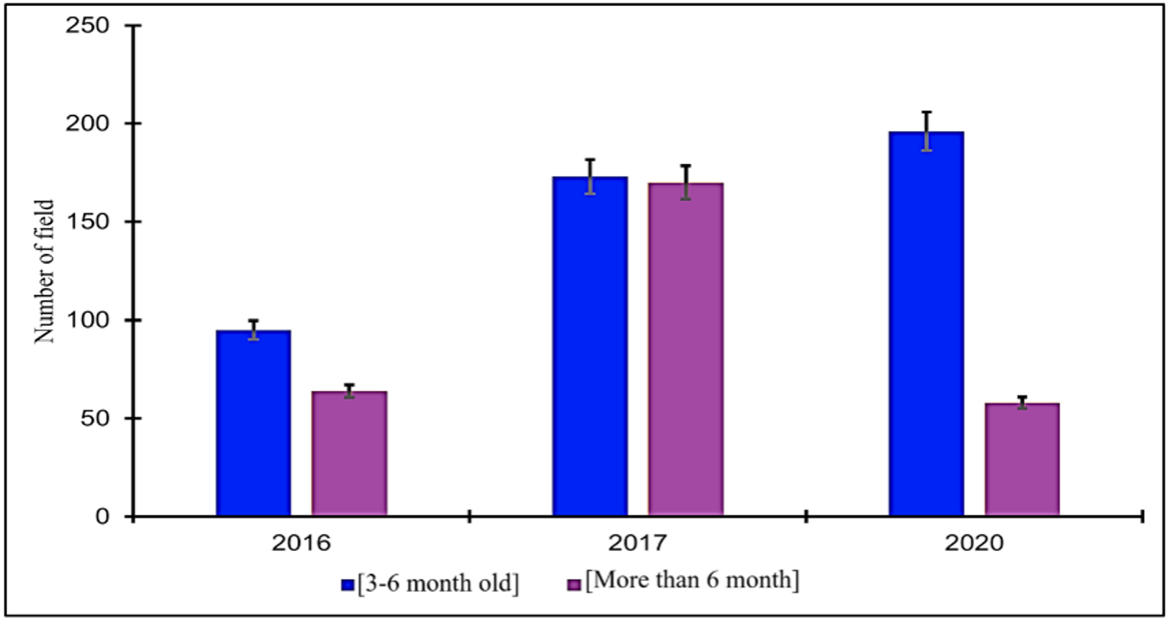
Crop age ranges according to agroecological zone over the surveyed

With regards to the crop age evaluated over a period of three years, the fields in 2016 and 2020 were predominantly classified as young (3 to 6 months old). In contrast, in 2017, the assessment revealed that half of the fields were between 3 and 6 months old, while the remaining half were over 6 months old (Fig. 7).

**Table 2:** Crops found in association with cassava according the agroecological zones

AEZs	Crops associated with cassava
AEZ I	*Elaeis guineensis* (Palm tree), *Hevea brasiliensis* (Rubber tree), *Musa paradisiaca* (Plantain), *Ipomoea batata* (Sweet potato), *Colocasia esculenta* (Taro), *Ananas comosus* (Pineapple), *Capsicum annuum* (Pepper), *Solanum melongena* (Eggplant)
AEZ II	*Elaeis guineensis* (Palm tree), Theobroma cacao (Cocoa), *Musa paradisiaca* (Plantain), *Ipomoea batata* (Sweet potato), *Colocasia esculenta* (Taro), *Capsicum annuum* (Pepper)
AEZ III	Theobroma cacao (Cocoa), *Musa paradisiaca* (Plantain), *Colocasia esculenta* (Taro), *Zea mays* (Maize), *Capsicum annuum* (Pepper), *Abelmoschus esculentus* (Okra)
AEZ IV	*Dioscorea spp.* (Yam), *Arachis hypogaea* (Peanut), *Capsicum annuum* (Pepper), *Abelmoschus esculentus* (Okra), *Solanum melongena* (Eggplant), *Solanum lycopersicum* (Tomato)
AEZ V	*Dioscorea spp.* (Yam), *Musa paradisiaca* (Plantain), *Arachis hypogaea* (Peanut), *Anacardium occidentale* (cashew nuts), *Capsicum annuum* (Pepper), *Zea mays* (Maize), *Abelmoschus esculentus* (Okra), *Solanum lycopersicum* (Tomato)
AEZ VI	*Zea mays* (Maize), *Sorghum bicolor* (Sorghum), *Ipomoea batata* (Sweet potato), *Anacardium occidentale* (cashew nuts), *Abelmoschus esculentus* (Okra)

### Effect of climatic factors on CMD associated epidemiological parameters

**Fig. 8:**
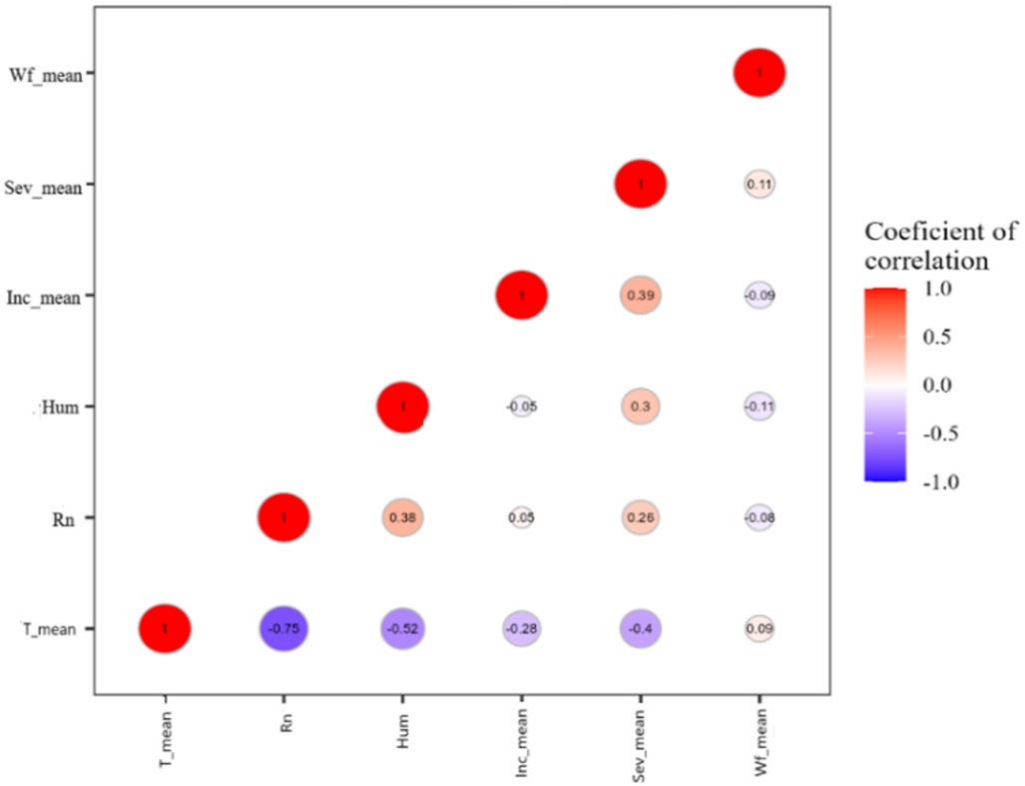
Spatiotemporal correlation matrix displaying correlation between climatic parameters: Temperature (T_mean), Rainfull (Rn) and Humidity (Hum) of January–December and epidemiological parameters: CMD incidence (Inc_mean), CMD severity (Sev_mean) and whiteflies abundance (Wf_mean) from strongly negative (dark blue) to strongly positive (dark red)

By using Pearson correlation coefficients for all variables, the observed relationship among CMD Incidence (Inc_mean), CMD symptom Severity (Sev_mean), Whitefly abundance (Wf_mean) and Temperature(T_mean), Rainfall (Rn) and Relative humidity (Hum) were validated for the PCA. These correlations were presented in correlation matrix of Fig. 8. All variables analyzed were found correlated with each other, some being more significant than others. Notably, the relationship between the Sev_mean and T_mean, Rn, Hum was statistically more significant than the Inc_mean with the T_mean. In addition, correlations were also found among the three CMD associated epidemiological parameter (CMD Incidence, CMD symptom Severity and Whitefly abundance), with a relatively significant correlation between Inc_mean and Sev_mean. However, no significant relationship was found between Wf_mean and the three climatic parameters on one hand and between Wf_mean and the two remaining CMD epidemiological parameters (Fig. 8).

**Fig. 9:**
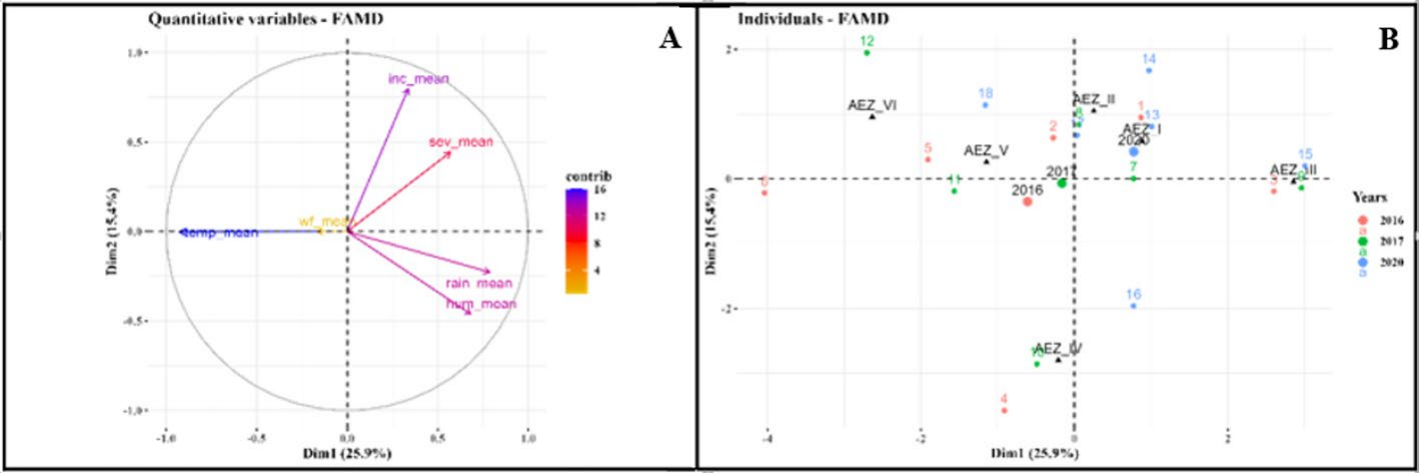
Factor analysis of mixed data (FAMD) Plots. **A** Biplot of the selected variables [temperature (T_mean), rainfull (Rn) and humidity (Hum) of January–December and CMD associated epidemiological parameters: CMD incidence (Inc_mean), CMD severity (Sev_mean) and whiteflies abundance (Wf_mean)]; Variables vector color according to their contribution in total variance: orange: low, blue: high. **B** Plot of individuals in relations to surveyed years and agroecological zones (2016: Red, 2017: Green, 2020: Blue)

Factor analysis of mixed data (FAMD) identified components 1 and 2 representing 41.3% of the variation among the variables analyzed. The first axis of the FAMD explained 25.9% and the second axis explained 15.4% of the overall variation. Axis 1 grouped variables analyzed into 2 clusters, regardless of the surveyed years (Fig. 9A). The cluster 1 is characterized by elevated temperatures and abundant whiteflies. In contrast, the second cluster is characterized by high CMD incidence, severe CMD symptoms, heavy rainfall and elevated relative humidity. Likewise, axis 2 sub-divides variables also into two distinct clusters. The first cluster of this axis is distinguished by severe CMD symptom (Sev_mean), the highest mean incidence (Inc_mean), abundant whiteflies (Wf_mean) and elevated temperatures (T_mean). The Second group included heavy rainfall (Rn) and high relative humidity (Hum).

The FAMD showed that temperature (T_mean) was the most important climatic variable affecting the uneven distribution of CMD between the AEZs (Fig. 9B). However, the contribution of Rainfall (Rn) and Relative humidity (Hum) was not negligible. Considering data sets for all 3 years combined and also the two axis, moderate negative correlation was demonstrated between temperature (T_Mean) and CMD incidence Inc_mean as well as with Severity mean (Sev_mean) and strong positive correlation was found between temperature (T_Mean) and abundance of whiteflies (Wf_mean). Moderate positive correlation was found between Rn and Inc_mean and also with Sev_mean. Rainfall and relative humidity were negatively correlated with CMD symptom severity (Sev_mean). Considering only CMD epidemiological parameters, strong positive correlation was found between Sev_mean and Inc_mean, while a weak negative correlation was identified between Wf_mean and both parameters (Fig. 9B). In general, the severity of symptoms was very pronounced in plots with high incidence.

### Effect of environmental factors on CMD associated epidemiological parameters

**Fig. 10:**
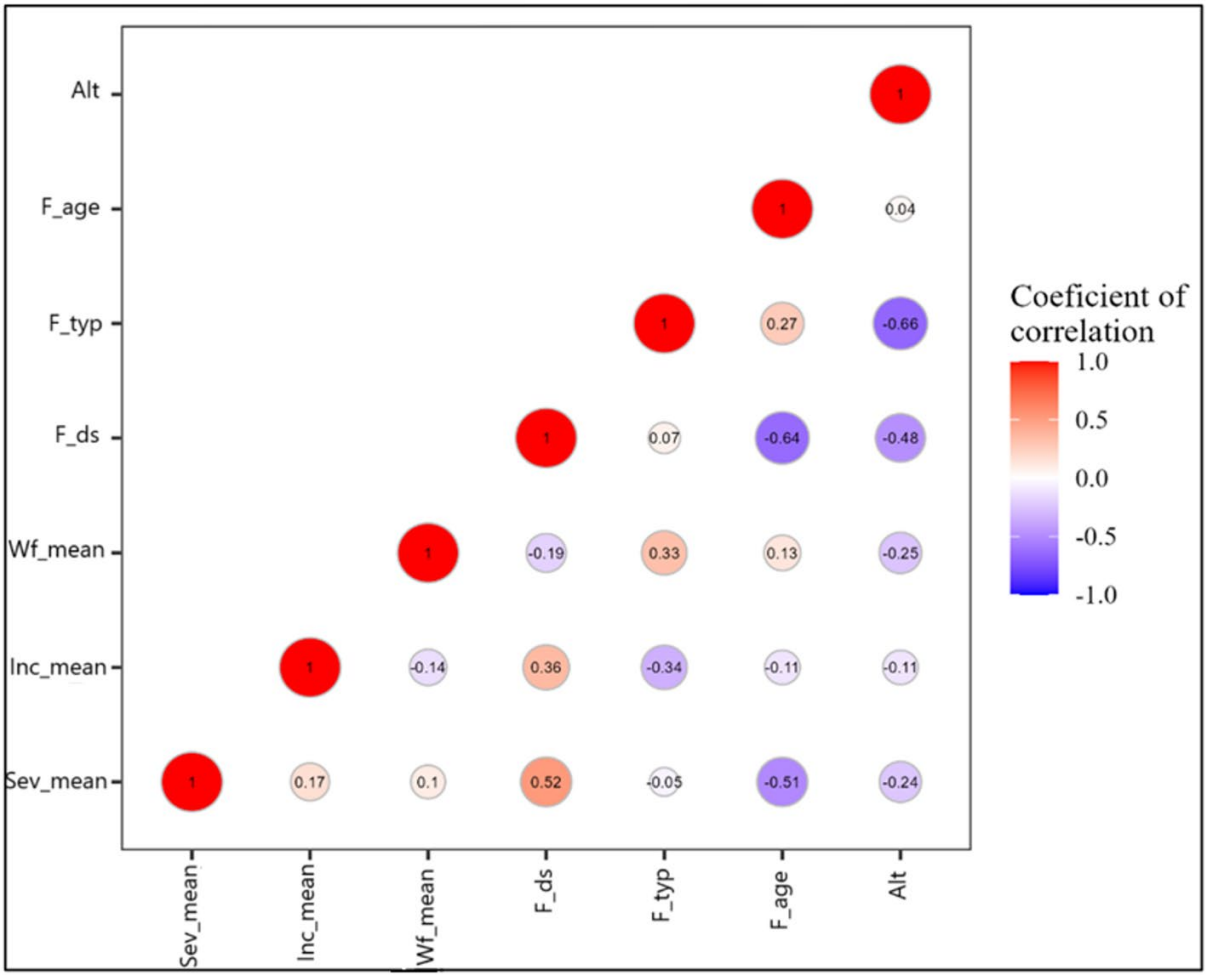
Spatiotemporal correlation matrix displaying correlation between environmental parameters: Field type (F_typ), Field age (F_age), Field density (F_den), Altitude (Alt) and epidemiological parameters: CMD Incidence (Inc_mean), CMD Severity (Sev_mean) and Whiteflies abundance (Wf_mean) from strongly negative (dark blue) to strongly positive (dark red)

The influence of altitude (Alt), Field density (F_den), Field type (F_typ), Field age (F_age) and their interactions with CMD incidence (Inc_mean), CMD symptom severity (Sev_mean) and Whitefly abundance (Wf_mean) was determined in Fig. 10. These environmental factors, acting in concert, contribute to the establishment of non-linear relationships between CMD associated epidemiological parameters. Indeed, F_den showed a significant correlation with Inc_mean and Sev_mean but not with Wf_mean. The CMD associated parameters, except Sev_mean, where also significantly correlated with F_typ while the only CMD associated epidemiological parameter correlated significantly with F_age was Sev_mean (Fig. 10). Regarding Alt, some correlations were found with Sev_mean and Wf_mean, although these relationships were not very significant.

**Fig. 11:**
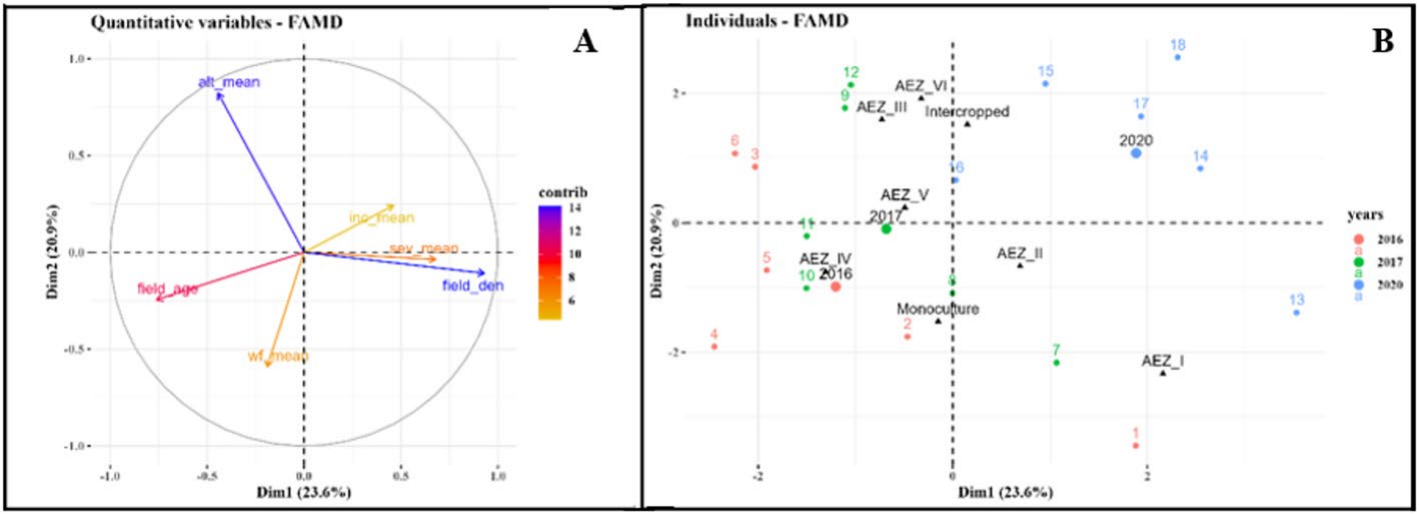
Factor analysis of mixed data (FAMD) Plots. **A**: Biplot of the selected variables [altitude (Alt), field density (F_Ds), field type (F_typ) and field age (F_age) and CMD associated epidemiological parameters: CMD incidence (Inc_mean), CMD severity (Sev_mean) and whiteflies abundance (Wf_mean). Variables vector color according to their contribution in total variance: orange: low, blue: high]; Variables color according to their contribution in total variance: orange: low, blue: high. **B**: Plot of individual in relations to surveyed years (2016: Red, 2017: Green, 2020: Blue)

For these data sets, factor analysis of mixed data (FAMD) identified components 1 and 2 as better representing the variation between the variables analyzed. These components as shown in Fig. 11A represented 44.5% of the variation. Axis 1 accounted for 23.6% of the overall variance and the second axis accounted for 20.9% of the total variance. Axis 1 grouped AEZs into two clusters. Cluster 1 is distinguished by abundant whiteflies (Wf_mean), young cassava fields (F_age) being in monoculture (F_typ) and highest altitudes (Alt). In contrast, the second cluster is characterized by high CMD incidence (Inc_mean), very severe symptoms (Sev_mean) and high cassava field density (F_den) being associated with other crops. Similarly, axis 2 sub-divides the AEZs into two distinct clusters. Cluster 1 is characterized by fields with the highest CMD incidence (Inc_mean) and in AEZ with high altitudes (Alt). In this group, more fields were found associated with other crops. However, the second group included the AEZs with high cassava field density (F_den) being in monoculture, very severe symptoms (Sev_m) and abundant whiteflies (Wf_mean). Young fields also characterize these zones (Fig. 11B).

The parameters that contributed most to variability between the AEZs were Alt, F_typ and F_den. They were the environmental parameters that play an important role in the variation of each of the different CMD associated epidemiological parameters observed among the AEZs. Considering data sets of all 3 years combined and also the two axis, a strong negative correlation was demonstrated between Alt and Wf_mean. Whiteflies were more abundant in zones with lower altitudes. Sev_mean and Inc_mean had respectively a moderate and weak negative correlation with Alt. Concerning F_den, it influenced very strongly Sev_mean, strongly the Inc_mean and moderately Wf_mean. These relationships were positives except F_den/ Wf_mean. Analysis showed that whitefly population was abundant in monoculture fields as these two parameters were positively correlated unlike CMD Inc_mean and Sev_mean which were more pronounced in cassava fields associated with other crops. The results also showed that Inc_m and Sev_mean were negatively correlated with F_age, the latter being positively correlated with Wf_mean (Fig. 11B).

### Cassava varieties grown in each agroecological zone

Cassava varieties largely cultivated in each agroecological zones were recorded in Table 3. The most common local varieties grown in almost all agroecological zones were Yacé, Bonoua and agba kangba. These varieties were described as more vulnerable to CMD. Agroecological zone I and V were zones with greater diversity of local varieties grown. Improved varieties were less cultivated in all the zones except agroecological zone IV where six different improved varieties (IM84, IM89, IM93, TMS 30572, Bocou 1, TME 693) were found. Except IM84, the others improved varieties found in this area are resistant to CMD. These results allow to deduct that most of cassava varieties cultivated in Côte d’Ivoire are vulnerable to CMD, which could induce important yields loss in farmer fields.

**Table 3:** Cassava varieties found in each agroecological zones

Agroecological zones	Local varieties	Improved varieties
AEZ I	Yacé, Bonoua, Tabote, Essakple, Accra banky, Brésil, Baéri	IM84, IM89, Bocou 1, Bocou 2
AEZ II	Agba kanga	TME 4/9
AEZ III	Bonoua, Yacé, Tabouka	TMS4(2)1425, Bocou 1
AEZ IV	Bahanin bouh, Bonoua, Yavo, Agba kanga, Yacé	IM84, IM89, IM93, TMS 30,572, Bocou 1, TME 693
AEZ V	Yacé, Bonoua, Yavo, Bahanin bouh, Agba kanga, Agba blé	IM84, TME 693, Bocou 1
AEZ VI	Bonoua, Diarassouba, Agba kanga	Bocou 1

## Discussion

The current work describes the correlation between CMD associated epidemiological parameters and environmental factors, on the one hand, and between these epidemiological parameters and the climatic factors, on the other hand. It is a comprehensive country-level (six agroecological zones in Côte d’Ivoire) study carried out during three years, 2016, 2017 and 2020. As the status of this disease demonstrated significant changes between years and agroecological zones [8], it was important to undertake this detailed study in order to understand this spatiotemporal dynamic of CMD epidemiological parameters.

Cassava varieties cultivated in the different agroecological zones might be related to the development of CMD over the years. In fact, local varieties have been found to be more widespread in almost all the localities visited, while they are known to be more susceptible to CMD [27]. This was the case of agroecological zones I and V which recorded greater diversity in the number of local varieties grown and where CMD had the greater incidence. However, in some few localities like agroecological zone IV, several improved cassava varieties were grown. Previous study has shown them to be tolerant or resistant to CMD [12]. It is not therefore surprising that CMD incidence was low in this part of the country.

Analysis showed a strong positive correlation between CMD incidence and the severity of the symptoms, while a weak correlation was identified between whitefly abundance and both parameters. The pathological explanation of the pronounced CMD symptoms in plots with high incidence is that in these plots (many individual plants with the disease), the disease had ample opportunity to spread and become more established, leading to an accumulation of viral load in infected plants and tissues being affected. This high level of infection allows more severe symptoms to manifest across the population because the pathogen has more hosts and sites to infect and exploit. [28–30] found similar results in Benin, Cameroon and Gabon respectively. The weak correlation between whitefly abundance and CMD incidence/severity, while seemingly counterintuitive given whiteflies’ primary role as vectors, highlights the multifactorial nature of CMD epidemiology. This finding does not refute their established role but rather suggests that the observed correlation might be influenced by time-lagged effects. Specifically, whitefly abundance data collected at 3–6 months may not align with the timing of initial virus transmission. Early infections (e.g., via infected cuttings) might dominate initial disease spread, with whitefly-driven secondary spread becoming significant at later stages. [8] have shown that cutting-transmitted infections predominated over those of whitefly-transmitted infections in Côte d’Ivoire during the three years of survey explaining this weak correlation between whitefly abundance and CMD incidence.

The spatial and temporal links among such CMD associated epidemiological parameters have been described by several studies [31–33]. Changes in these parameters have been first attributed to climate conditions, which seems to be different between the zones surveyed. According to [34, 35], climatic conditions can facilitate the establishment of pests and pathogens (including viruses and their vectors) into previously unsuitable regions.

In the current study, we analyzed the three main climatic factors that were suspected to have influenced the variations in CMD associated parameters between agroecological zones. It was found that they also varied significantly across the surveyed zones. Unlike the CMD associated epidemiological parameters, which showed significant changes both between agroecological zones and between years, the climatic parameters analyzed varied only between the AEZs but not over the years. Therefore, only the spatial changes in the epidemiological parameters associated with CMD can be linked to climatic factors, but not the temporal changes.

Using the spearman correlation test combined with multivariate analysis, our study showed relationships between some of the climatic parameters analyzed and CMD associated epidemiological parameters. Out of the three climatic parameters analyzed, temperature was the one that mostly affected the uneven distribution of CMD between the AEZs. In this study, a significant moderate negative correlation was observed between CMD incidence and temperature on one side and between the severity of CMD symptoms and temperature on the other side. This means that low temperatures are favorable to the spread of CMD and contribute to enhanced disease severity. According to [36], leaves produced during periods of cool weather tend to be affected more than those produced under hotter conditions. This might, at least partially, explain why the incidence of CMD was found to be higher in the southern (AEZ I and AEZ II) and western parts (AEZ III) of the country, where temperatures are generally lower. In contrast to what was found in this study, [37] found strong positive relationships between Cassava Brown Streak Disease (CBSD) incidence and temperature in Tanzania. Likewise, heavy rainfall and humid climate also contribute enhancing disease symptoms in Côte d’Ivoire since the results showed a strong positive correlation between these parameters. High humidity supports the growth of the whitefly vector, which transmits the virus, and heavy rainfall can influence its spread and impact plant health. It is therefore not surprising that the zones with highest rainfall (agroecological zones I and III in the southern and western Côte d’Ivoire respectively) were among the most susceptible to the disease during the three years of surveys.

By analyzing the four targeted environmental parameters, we noticed that the parameters that contribute most to variability among the AEZs are Altitude, field density per zone and cropping system (field type). These environmental factors, acting synergistically, highly contribute to the variability of epidemiological parameters associated with CMD between agroecological zones over years. Altitude significantly influenced whitefly abundance, CMD symptom severity and weakly influenced CMD incidence. Indeed, it was negatively correlated with all three CMD-associated epidemiological parameters. This means that whiteflies are more abundant in fields with severe CMD symptoms at lower altitudes than at higher altitudes. This situation could be explained by the fact that whiteflies cannot fly easily at very high altitudes because reduced air density makes it harder to generate lift with their wings, while low oxygen levels hinder their metabolism and muscle function. Therefore, they are restricted to low-altitude regions in order to proliferate. In addition, the pronounced severity of the disease symptoms at low altitudes implies that the vector’s activity in transmitting CMD is more intense in these areas. Their synergy with the use of infected cutting would contribute to accentuating the symptoms caused by the disease. Similar reports were made in Tanzania and Madagascar [18, 30, 33, 37]. The regression analysis also revealed a strong positive relationship between the incidence of CMD, symptoms severity and field density. A previous study found that CMD affect more cassava fields in the southern and western Côte d’Ivoire [8]. These regions constitute the area of high cassava production, characterized by elevated planting densities. This observation provides a compelling rationale for the observed positive correlation between these parameters. These findings are further corroborated by similar findings from several studies that have confirmed spatial associations between cassava field density per zone and the development of the disease [38]. The current study also demonstrated that field density has a negative effect on whitefly abundance, even if this relationship was weak. However, some studies showed evidence of massive increases in whitefly populations, particularly of *Bemisia tabaci* species, largely in areas of intensive cassava cultivation [39–41]. Changes in whitefly abundance from year to year between agroecological zones might have been partly due to cropping system, which was found to be strongly correlated with it. Indeed, these two parameters were positively linked. These results are corroborated by similar work carried out by several authors [9] in Côte d’Ivoire; [17] in Cameroon; [42] in India who confirmed the link between cropping systems and whiteflies abundance. The abundance of whitefly populations in cassava monoculture system demonstrated in this study can be attributed to the absence of competition from other crops in these fields. However, the presence of one or more other crops in association with cassava creates competition between the crops in terms of hosting these whiteflies. In monoculture systems, when the whiteflies need to feed, the cassava leaf represents the sole available option, thereby contributing to the substantial population sizes observed on plants. Whiteflies are one of the sources of the spread of CMD. Their limited presence in intercropped fields would suggest that this cropping system is the most suitable for the control of cassava mosaic disease. Curiously, our data revealed a negative correlation between cropping system and CMD incidence. The disease incidence was found lower in monoculture systems than in intercropped systems. The crops associated with cassava were probably not suitable to contribute reducing the disease, because some intercrops act as alternative hosts for the vector hence increasing whitefly population, disease spread, viral titre and CMD severity in the field, as previous studies mentioned [1, 17, 43]. While some intercrops can increase disease pressure by attracting more whiteflies, others can reduce CMD by lowering whitefly populations or creating barriers. This is the case with intercropping systems involving cassava/maize and/or cassava/cowpea, which, according to these authors, have beneficial effects on reducing the incidence of CMD. This study showed that the association cassava/Plantain was more found in AEZ I and AEZ II where CMD incidence was high while cassava/Maize were more encountered in AEZ V and AEZ VI. A low incidence of CMD was observed in these areas, thereby confirming that this specific cropping system was more effective in reducing the impact of the disease.

Crop age was one of the parameters analyzed which contributed moderately to the variability of CMD associated parameters between zones. The results also showed that CMD symptoms severity were strongly negatively correlated with field age. This suggests that young cassava fields are more susceptible to CMD with more severe symptoms than older fields. The physiological basis for severe symptoms observed in young plants is that they exhibit a high degree of metabolic activity. It is precisely this attribute that facilitates the rapid proliferation of the virus within the plant organism.

## Conclusion

This study aimed to understand changes in CMD epidemiological parameters from year to year in the study area. Climatic and non-climatic factors interact with each other to influence the disease development. The current study revealed that altitude and planting density have a greater influence on whitefly abundance and CMD severity in each agroecological zones. It also showed that, temperature, was by far the most important climatic factor that significantly influenced the variations in CMD incidence in the study area. One of the key factors influencing the epidemiology of CMD in Côte d’Ivoire is the use of susceptible local cassava varieties in almost all agroecological zones of the country. These results provide a basis for understanding the epidemiology of CMD. They emphasize the need to consider climatic and non-climatic factors when developing integrated control measures against CMD and educate farmer to the use of clean cuttings as well as choosing the right planting system.

## Data Availability

Data will be available upon request.
